# Ultrasensitive detection of lipoarabinomannan with plasmonic grating biosensors in clinical samples of HIV negative patients with tuberculosis

**DOI:** 10.1371/journal.pone.0214161

**Published:** 2019-03-26

**Authors:** Aaron Wood, Syed Barizuddin, Charles M. Darr, Cherian J. Mathai, Alexey Ball, Kyle Minch, Akos Somoskovi, Beston Hamasur, John T. Connelly, Bernhard Weigl, Alfred Andama, Adithya Cattamanchi, Keshab Gangopadhyay, Sangho Bok, Shubhra Gangopadhyay

**Affiliations:** 1 Department of Electrical Engineering and Computer Science, University of Missouri, Columbia, Missouri, United States of America; 2 Intellectual Ventures Laboratory, Bellevue, Washington, United States of America; 3 Intellectual Ventures’ Global Good Fund, Bellevue, Washington, United States of America; 4 Biopromic AB, Solna, Sweden; 5 Department of Microbiology, Tumor and Cell Biology (MTC), Karolinska Institutet, Stockholm, Sweden; 6 College of Health Sciences, Makerere University, Kampala, Uganda; 7 Division of Pulmonary and Critical Care Medicine, University of California San Francisco; 8 Zuckerberg San Francisco General Hospital, San Francisco, California, United States of America; 9 Curry International Tuberculosis Center, University of California San Francisco, San Francisco, California, United States of America; 10 Department of Engineering and Technology, Southern Utah University, Cedar City, Utah, United States of America; Consiglio Nazionale delle Ricerche, ITALY

## Abstract

**Background:**

Timely diagnosis of tuberculosis disease is critical for positive patient outcomes, yet potentially millions go undiagnosed or unreported each year. Sputum is widely used as the testing input, but limited by its complexity, heterogeneity, and sourcing problems. Finding methods to interrogate noninvasive, non-sputum clinical specimens is indispensable to improving access to tuberculosis diagnosis and care. In this work, economical plasmonic gratings were used to analyze tuberculosis biomarker lipoarabinomannan (LAM) from clinical urine samples by single molecule fluorescence assay (FLISA) and compared with gold standard sputum GeneXpert MTB/ RIF, culture, and reference ELISA testing results.

**Methods and Findings:**

In this study, twenty sputum and urine sample sets were selected retrospectively from a repository of HIV-negative patient samples collected before initiation of anti-tuberculosis therapy. GeneXpert MTB/RIF and culture testing of patient sputum confirmed the presence or absence of pulmonary tuberculosis while all patient urines were reference ELISA LAM-negative. Plasmonic gratings produced by low-cost soft lithography were bound with anti-LAM capture antibody, incubated with patient urine samples, and biotinylated detection antibody. Fluorescently labeled streptavidin revealed single molecule emission by epifluorescence microscope. Using a 1 fg/mL baseline for limit of detection, single molecule FLISA demonstrated good qualitative agreement with gold standard tests on 19 of 20 patients, including accurately predicting the gold-standard-negative patients, while one gold-standard-positive patient produced no observable LAM in urine.

**Conclusions:**

Single molecule FLISA by plasmonic grating demonstrated the ability to quantify tuberculosis LAM from complex urine samples of patients from a high endemic setting with negligible interference from the complex media itself. Moreover, agreement with patient diagnoses by gold standard testing suggests that single molecule FLISA could be used as a highly sensitive test to diagnose tuberculosis noninvasively.

## Introduction

Estimates vary, but recent work suggests that greater than one quarter of incident tuberculosis (TB) cases went undiagnosed or unreported in 2018 [1]. Timely access to diagnosis is critical for disease management; however, several disease-intrinsic and engineering challenges compromise the development of rapid, near-patient, diagnostic tests. Sputum is a widely-used input for diagnosis, but the complexity and heterogeneity of this sample matrix require specialized equipment and/or infrastructure, thus, limiting time-to-detection (TTD) and point-of-care (POC) deployment. A recent example is the rollout of the PCR-based GeneXpert MTB/RIF test, which incorporates a largely automated workflow from patient sputum sample to result and TTD is only a few hours. However, GeneXpert is relatively expensive (≥$10 per test after hardware) and requires operator training, instrument upkeep, and calibration [2]. Furthermore, not all patients have TB disease that leads to sputum production, including children and HIV positive individuals, making timely diagnosis of these populations particularly challenging [3–5]. Reducing reliance on sputum, utilizing alternative sample sources, considering alternative TB and TB disease biomarkers, assay simplification, and decreasing cost are all important parameters that will lead to novel diagnostics that inform an affordable POC test.

Lipoarabinomannan (LAM) is a prominent glycolipid of the mycobacterial cell wall that has received substantial attention as a TB disease-associated biomarker [6–8]. LAM can be detected from sputum, blood, and urine samples, and can be detected in routine immunologic tests such as enzyme-linked immunosorbent or lateral flow assays (ELISA or LFA, respectively) [9, 10]. To this, the Alere Determine TB LAM Ag Test [Abbott Diagnostics, Santa Clara, CA, USA] is a commercially available rapid LFA LAM diagnostic test. While demonstrating the feasibility of LAM as a TB biomarker, the sensitivity/specificity profile led the World Health Organization (WHO) to limit its endorsement to a subgroup of HIV+ adult in-patients with advanced disease and CD4+ T-cell counts <100 cell/μL and signs and symptoms of active TB disease [11]. Other recently published studies on LAM detection assays report relatively high limits of detection (LOD), between 10–0.05 ng/mL, which still may not enable reliable, routine TB diagnosis regardless of HIV and CD4 status [12, 13].

Increasing sensitivity/lowering the LOD is a promising avenue for assessing whether or not LAM could serve as a more broadly useful TB biomarker. One approach generating a measure of success at improving LODs and clinical performance is to capture LAM from larger volumes [14, 15]. Others report an electrochemiluminescence-based ELISA format to achieve LODs in a similar range, 350–650 fM (6–11pg/mL), and demonstrated the ability for that assay to successfully detect LAM in patient urines [8]. While this is >3 logs more sensitive than standard LFA/ELISA methods, we hypothesized that additional increases in sensitivity by enhancing signal-to-noise ratio (SNR) would permit the assessment of LAM as a TB diagnostic biomarker from clinically relevant samples. In the present work, we describe a fluorescence-enhancing plasmonic grating platform combined with a fluorescence-linked immunosorbent assay (FLISA) for LAM detection capable of detecting near single molecule (10 fM) LAM concentrations from clinical samples. Plasmonic gratings are made of a series of nanoscale ridges and grooves, typically of noble metals, which can convert incident photons into standing electromagnetic (EM) waves at the surface of the metal film in a process known as surface plasmon resonance (SPR) [16–18]. These EM waves can interact with and excite nearby dipolar molecules, such as fluorescent molecules. These molecules can, in turn, transfer energy nonradiatively to the grating to form a radiative plasmon, which is emitted from the grating at a specific angle and wavelength. This phenomenon, surface plasmon coupled emission (SPCE), can increase the observed emission intensity by up to 200× the intensity of the same fluorescent molecules on a non-plasmonic substrate [19–21]. The increased emission intensity produces intrinsic signal amplification that greatly enhances the SNR of fluorescence images and, paired with a fluorescent detection mechanism, enables much lower LODs, including single molecule detection [21–23]. One recent report described a FLISA for LAM from buffered saline with LODs in the range of 1 fM (~17.5 fg/mL) [24]. The objective of the present work was to demonstrate that the sensitivity enhancement provided by the plasmonic grating platform would enable detection of LAM in urine samples collected from HIV-negative, TB-confirmed patients that was undetectable by our conventional reference ELISA and to determine LAM concentrations in these samples. Our results suggest that the use of TB LAM as a diagnostic biomarker requires detection into this clinically relevant low femtomolar range.

## Materials and methods

### Study population and setting

Patient urine samples were collected between July 1, 2017 and January 31, 2018 as part of an ongoing study of non-sputum-based biomarker tests for TB diagnosis at Kiruddu General Referral Hospital, Makindye Division, Kampala, Uganda. All study procedures were reviewed and approved by the Makerere University School of Medicine Research Ethics Committee, the University of California, San Francisco Committee on Human Research, and the Uganda National Council for Sciences and Technology. All enrolled patients were provided written informed consent after having read the approved consent form or having said form read to them. The consent form was available in both English and Luganda and the study coordinator overseeing informed consent was fluent in both languages.

Hospitalized and outpatient adults who submitted a sputum sample for GeneXpert MTB/RIF testing were included in the study. Patients were excluded if (1) Xpert results were indeterminate; (2) they were presently taking or had taken in the past 12 months anti-TB treatment or agents with anti-TB activity (e.g. fluoroquinolones); or (3) they refused or were unable to provide informed consent. In addition, for this proof-of-principle sub-study, we selected only HIV-negative patients (since TB LAM has been shown to be more difficult to detect in this population [11, 25, 26]) whose urine samples were negative for LAM with our reference ELISA method.

### Urine sample collection

Urine samples were collected upon enrollment, de-identified, and kept on ice for no more than 3 hours before several aliquots were frozen at -80 °C. Subsequently, frozen aliquots were shipped on dry ice to Intellectual Ventures Laboratory (IVL) for testing by a reference ELISA developed at IVL and transferred to collaborators for testing by SM FLISA. IVL staff blinded collaborators as to the ELISA results and the TB diagnosis of the patients associated with the urine specimens evaluated by SM FLISA and had no involvement in performing SM FLISA assays or analyzing the resulting data.

### Reference testing by GeneXpert direct molecular testing and growth detection

GeneXpert MTB/RIF (Xpert) was used in combination with culture as the gold standard for diagnosing active pulmonary TB disease. Patients provided sputum samples for testing by GeneXpert and culture before initiation of anti-tuberculosis therapy [27]. A positive Xpert result was considered a conclusive TB diagnosis. If Xpert was negative, the patient provided 2 additional spot expectorated sputum samples for growth detection in the Mycobacterium growth indicator tube 960 (MGIT) system (Becton-Dickinson Microbiology Systems, Sparks, MA) and on solid (Lowenstein Jensen) culture [28, 29]. A convenient sample of 20 specimens stored at -80 °C was selected for the proof-of-principle experiments.

### Reference ELISA protocol

Standard sandwich ELISA was used to assess LAM concentration in urine specimens. Briefly, Nunc Maxisorp 96 well ELISA plates were coated with 5 μg/mL anti-LAM capture antibody, clone BPM102 (Biopromic AB, Solna, Sweden), in carbonate coating buffer for 1 hour at room temperature. Wells were washed 5 times each in phosphate buffered saline (PBS) with 0.05% Tween-20 surfactant then blocked with 1.0% w/v bovine serum albumin (BSA, Sigma fraction—V) in PBS for 1 hour at room temperature. After washing again as above, patient urine samples and a standard curve of purified LAM (Biopromic AB, Solna, Sweden) spiked in healthy control urine (1000–31 pg/mL) were added to the wells for an overnight incubation at 4 °C. Wells were washed as above and incubated with 0.3 μg/mL biotinylated (~8 biotins per antibody) anti-LAM detection antibody, clone BPM101 (Biopromic AB, Solna, Sweden) in PBS with 0.5% w/v BSA for 1 hour at room temperature. Wells were washed again and incubated with high sensitivity streptavidin-HRP (Pierce) at 1:7500 in PBS. Wells were washed again and exposed to TMB (high sensitivity by Thermo) for 5 minutes at room temperature. The colorimetric reaction was stopped by the addition of 0.5 N HCl. A450 and A550 values were measured on a spectrophotometer. Corrected A450 values (A550 subtracted from A450 values) were determined for each data point.

### Plasmonic grating fabrication

**Fig 1:**
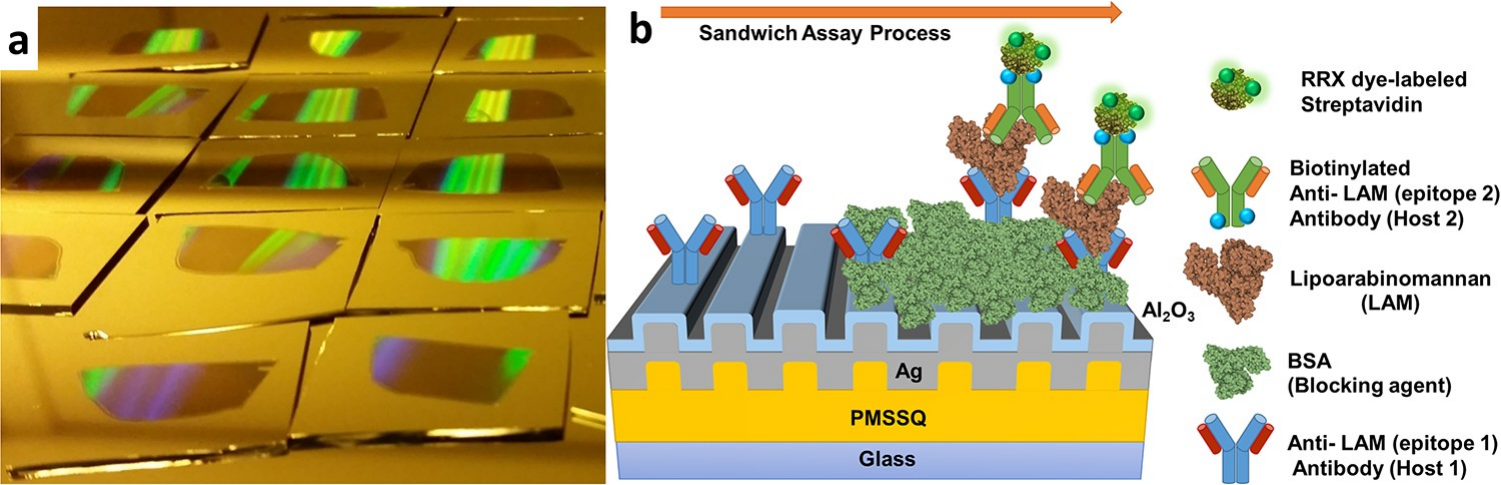
(a) A photographic image of fabricated PMSSQ gratings on 1 inch x 1inch glass slides and (b) a diagram of FLISA on gratings showing the sandwich structure of antibodies for detection of LAM.

Plasmonic grating platforms were fabricated (Fig 1a) by soft lithography as described in detail previously [21, 30–33].

Briefly, a commercially available HDDVD was cleaned and molded with polydimethylsiloxane (PDMS, 5:1 Sylgard 184, Gelest). Plain glass slides (Corning) were bath sonicated in acetone, methanol, and deionized water (18.2 MΩ-cm), dried with flowing nitrogen, then soaked in Piranha Solution (3:1 H_2_SO_4_:H_2_O_2_) for 10 minutes, rinsed in copious deionized water, and dried in flowing nitrogen. Cured PDMS stamps were cut to size and spin-coated with a 3% polymer ink (GR650F polymethylsilsesquioxane (PMSSQ), Techneglas) in ethanol and pressed onto the glass slides. The nascent PMSSQ gratings were crosslinked by vapor treatment with 3-aminotriethoxysilane (APTES) in ethanol under vacuum for 1 hour, pre-baked at 60 °C for 3 hours, then baked at 400 °C for 1 hour. The gratings were metallized with 100 nm silver by AJA RF Magnetron sputter and finally coated with 10 nm alumina by low-temperature (65 °C) atomic layer deposition (ALD) in a method similar to [34].

### Plasmonic grating FLISA surface preparation

Immediately prior to antibody binding, alumina-coated plasmonic gratings were exposed to 30 s 7 W CO_2_ plasma in a plasma-enhanced chemical vapor deposition (PECVD) chamber to generate carboxyl and carbonyl groups on the alumina surface. Activated gratings were then topped with a ProPlate 24 well slide adapter (Grace Bio-Labs) using custom 3D printed clips for easy removal during imaging. Activation buffer consisting of 75 μL pH 6.0 2-(N-morpholino)ethanesulfonic acid (MES) with 11 mg/mL sulfo-N-hydroxysuccinimide (Sulfo-NHS) and 4 mg/mL 1-ethyl-3-(3-dimethylaminopropyl)carbodiimide (EDC) was added to each well and allowed to sit at room temperature for 10 minutes. Anti-LAM capture antibodies (BPM102) were diluted to 20 μg/mL in 75 μL pH 8.0 MES, added to each well, and incubated overnight at 4 °C. All wells were washed by shaking three times for 5 minutes apiece with pH 7.4 phosphate buffered saline (PBS) with 0.1% Tween-20 surfactant (PBS-T). The final PBS-T solution was replaced with 150 μL of PBS with 3% w/v bovine serum albumin (BSA) for 1 hour to block any open binding sites to the sensor surface. Gratings were again washed 3× with PBS-T and ready for SM FLISA assay.

### Single molecule (SM) FLISA on plasmonic gratings

SM FLISAs (Fig 1b) were performed in the manner of [33] on blinded patient urine samples divided at random into two groups, one of 8 and one of 12, to accommodate the large number of samples. Vials of patient urine samples were thawed and vortexed, then deposited into different wells in 100 μL aliquots (n = 3 per patient) and incubated for 2 hours at 4 °C. Each group also contained a control set of LAM-spiked purified human urine with eight concentrations from 1 fg/mL to 10 ng/mL. Sample-inoculated gratings were washed 3× with PBS-T as above and 100 μL of PBS with 5 μg/mL biotinylated anti-LAM detection antibodies (BPM101) was added to each well and left to incubate for 2 hours at 4 °C. Gratings were again washed 3× with PBS-T and 100 μL of PBS with 10 μg/mL AlexaFluor 568-labeled Streptavidin was added to each well and left to incubate for 2 hours at 4 °C. All samples were again washed 3× with PBS-T and buffer replaced with plain PBS. Finally, buffer was replaced with 100 μL of deionized water and the slide modules were detached from the slide. Large area (1 in × 1.5 in) coverslips were placed over the slides and remaining water. Samples were then imaged using an ORCAFlash 2.8 CMOS camera at 5 s integration time on a BX51WI Olympus epifluorescence microscope using a 60× water-immersion objective. The acquired images were analyzed by using ImageJ to quantify the LAM molecules. The images exhibited blinking fluorescence spots indicating single molecules or near single molecules that was counted (S1 Fig).

## Results

As shown in Table 1, 18 of the 20 samples were Xpert positive, while the other two (P50 and P159) were confirmed free of pulmonary TB disease by the combination of negative Xpert and subsequent culture results. SM FLISA method showed <1 fg/mL for both negative patient samples while a conventional ELISA method was not able to measure quantitatively. The LAM concentrations in the patient samples were calculated relative to the corrected A450 values of the standard curve with known spiked LAM concentrations. All samples tested did not show any sign of LAM from ELISA while SM FLISA demonstrated quantified LAM for all samples which agreed with the results from Xpert (Table 1).

**Table 1:** Comparison of TB diagnostic results by various methods. A conventional ELISA was not able to quantify LAM concentration while SM ELISA showed not only the quantification of LAM but also the agreement with Xpert results. SM FLISA 1 and 2 indicated two independent assays sets.

Patient No.	Xpert			Culture		ELISA	SM FLISA
	MTB	SQ	RIF	MGIT	LJ	[LAM] (pg/mL)	[LAM] (pg/mL)
50	-	-	-	-	-	0	<0.001^1^
89	+	++++	NR	ND	ND	0	11.59 ± 9.85^1^
119	+	++	NR	ND	ND	0	8340 ± 6900^2^
124	+	+++	NR	ND	ND	0	3.40 ± 2.06^2^
128	+	+	I	ND	ND	0	0.301 ± 0.243^1^
130	+	++	NR	ND	ND	0	0.783 ± 0.300^1^
146	+	+++	NR	ND	ND	0	>10000^2^
149	+	++	NR	ND	ND	0	18.78 ± 17.52^1^
159	-	-	-	-	-	0	<0.001^1^
160	+	++++	NR	ND	ND	0	0.229 ± 0.158^2^
163	+	+	NR	ND	ND	0	0.372 ± 0.206^1^
183	+	++	NR	ND	ND	0	0.012 ± 0.008^2^
187	+	++++	NR	ND	ND	0	0.0133 ± 0.0096^2^
199	+	++++	NR	ND	ND	0	0.00615 ± 0.0059^2^
205	+	++++	NR	ND	ND	0	1.263 ± 0.875^2^
206	+	++++	NR	ND	ND	0	8910 ± 6240^2^
213	+	++++	NR	ND	ND	0	<<0.001/Blank^2^
214	+	+++	NR	ND	ND	0	5760 ± 4000^2^
215	+	+	NR	ND	ND	0	123.7 ± 67.1^1^
220	+	++	NR	ND	ND	13.01	1.804 ± 1.394^2^

MTB = Mycobacterium tuberculosis, SQ = Semi-Quantitative, RIF = resistance to rifampicin, MJIT = Mycobacteria Growth Indicator Tube, LJ = Löwenstein–Jensen medium culture, NR = No Resistance, I = Indeterminate Resistance, ND = Not Done,

^1^SM FLISA Assay 1,

^2^SM FLISA Assay 2

**Fig 2:**
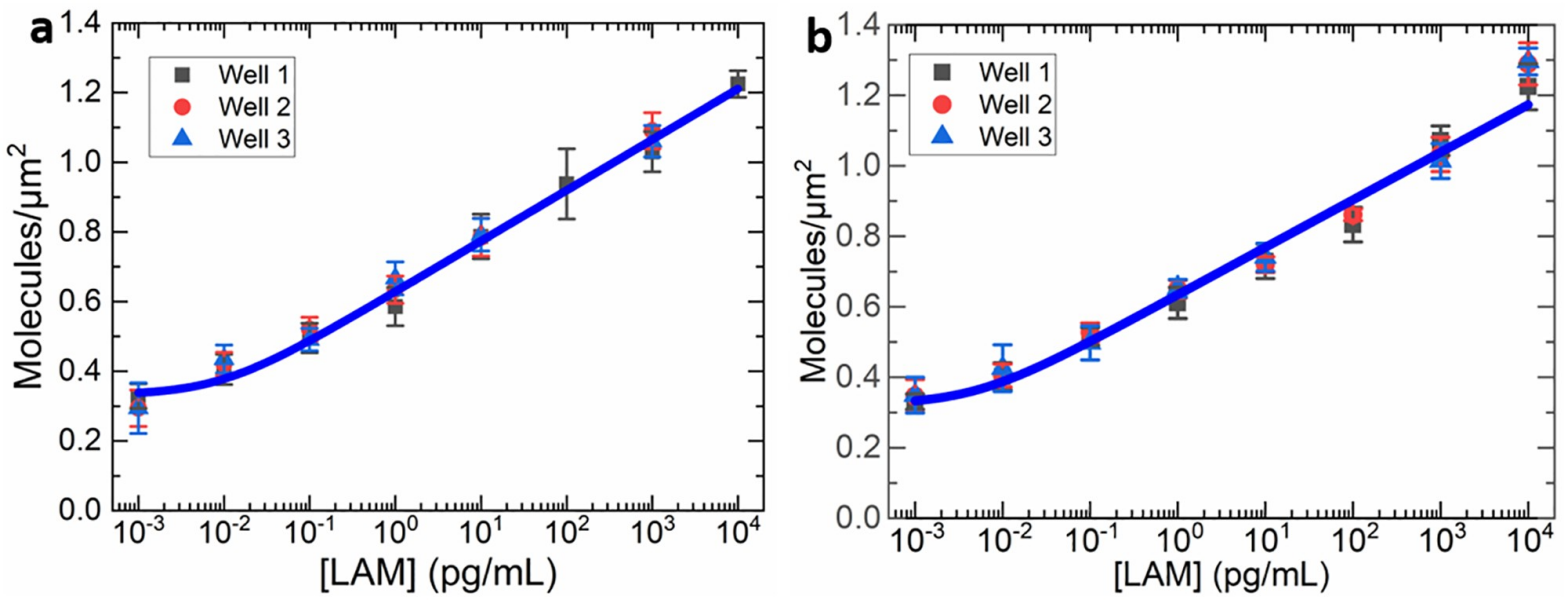
SM FLISA standard curves and fitted data for (a) Assay #1 and (b) Assay #2 demonstrating a good agreement with each other for reproducibility of the data.

Single molecule blinking behavior was observed for all samples and all but one of the control and patient samples required a CMOS integration time of 5 s. The number of LAM molecules located within a 6 × 6 μm^2^ grid of the plasmonic sensor surface were counted by observation of single molecule blinking behavior over the course of a several minute time trace. Counts were averaged across 12 grids per well and 3 wells per patient and plotted against the LAM standard concentration curve generated by similar analysis of the spiked purified urine samples (S1 Fig). Within the eight concentrations of each group control set, a transition from bulk fluorescence emission to SM emission behavior was observed at ~1 ng/mL LAM concentration. Fig 2 shows the average number of LAM molecules observed per square micrometer of sensor surface.

Overall, a significant difference (95% confidence interval) was obtained between samples with 2-orders of magnitude difference in LAM concentration (e.g. 1 fg/mL and 100 fg/mL), but not with 1-order of magnitude difference in LAM concentration. A log-linear function was found to best fit the data from each group and equations generated to provide a standard curve for each group with adjusted R^2^ = 0.994 and 0.9844, respectively (Table 2). The curve coefficients for each group control set are in good agreement despite the tests being performed roughly 1 week apart and on different grating substrates, an important validation of the consistency of the grating sensor surface and the reproducibility of the SM counting method to determine LAM concentration. These equations were later used to translate the number of molecules per grid of the patient samples into corresponding LAM concentrations.

**Table 2:** Fit Parameters for LAM Concentration Standard Curves representing the reproducibility of SM FLISA.

Fitted Equation: Y = A − B* ln(X+C) Molecules per μm^2^ (Y) vs [LAM] (X)				
	A	B	C	R^2^
**Assay #1**	0.628 ± 0.016	-0.063 ± 0.003	0.009 ± 0.006	0.996
**Assay #2**	0.634 ± 0.017	-0.056 ± 0.004	0.005 ± 0.004	0.989

**Fig 3:**
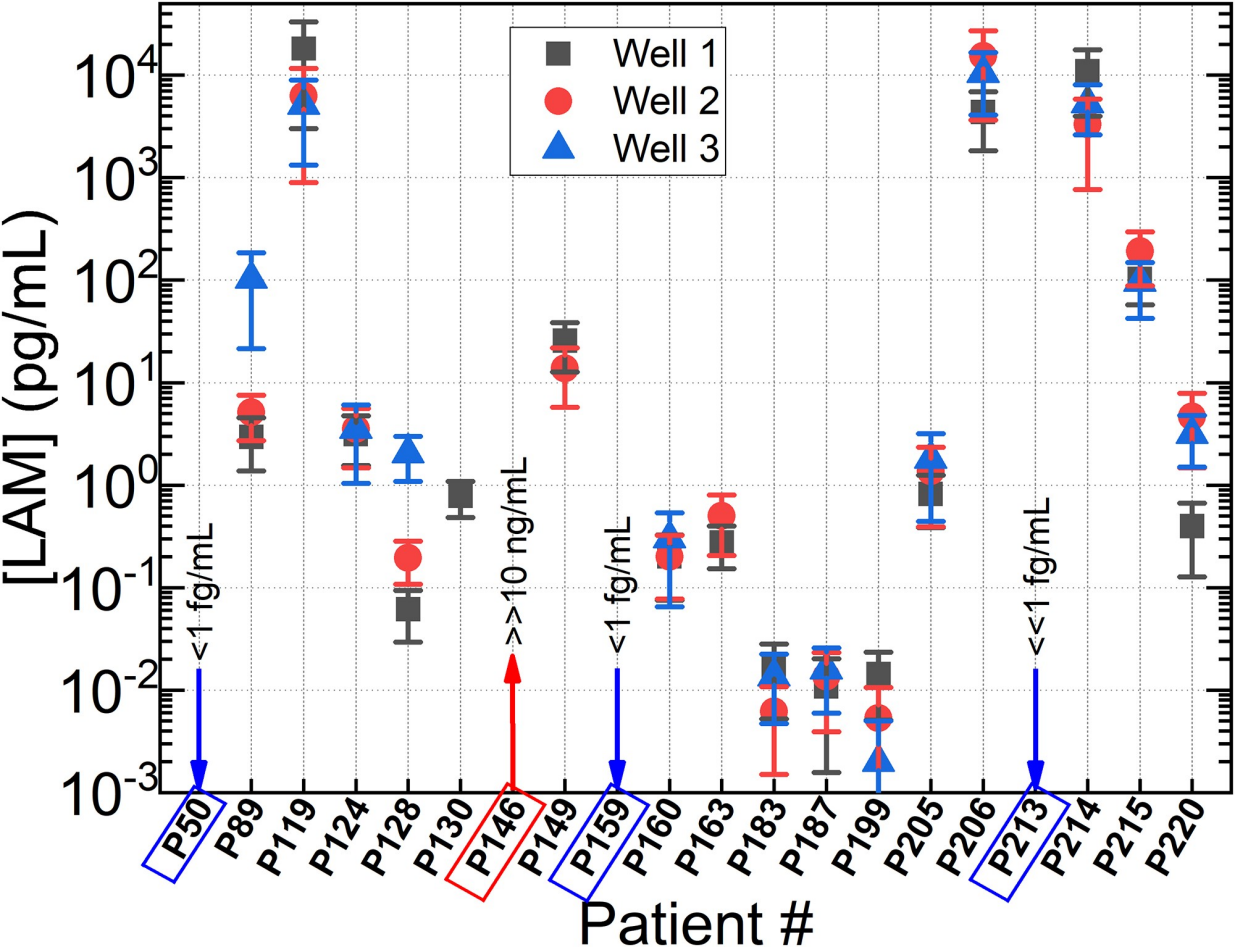
Combined result graph of all 20 patient samples with three measurement for each patient. The result demonstrated good agreement with Xpert while conventional ELISA could not quantify LAM concentration.

Patient sample SM count data and calculated LAM concentration are summarized in Table 3 and LAM concentration plotted in Fig 3.

**Table 3:** Combined SM FLISA Data representing the actual counts of single molecules per unit area and the corresponding conversion to concentration in pg/mL.

Patient #	Molecules per μm^2^	[LAM] (pg/mL)
**Assay #1**		
**50**	0.296 ± 0.0572*	< 1 fg/mL (Not Blank)
**89**	0.783 ± 0.1197	11.59497 ± 9.85478
**128**	0.554 ± 0.0973	0.30056 ± 0.24333
**130**	0.613 ± 0.0301	0.78333 ± 0.30013
**149**	0.814 ± 0.1707	18.77916 ± 17.52481
**159**	0.244 ± 0.0594	< 1 fg/mL (Not Blank)
**163**	0.567 ± 0.0491	0.37196 ± 0.20578
**215**	0.933 ± 0.0494	123.71183 ± 67.07588
**Assay #2**		
**119**	1.163 ± 0.103	8338.799 ± 6900.996
**124**	0.706 ± 0.055	3.401 ± 2.064
**146**	>> 10 ng/mL	>> 10 ng/mL
**160**	0.549 ± 0.066	0.229 ± 0.158
**183**	0.394 ± 0.041	0.012 ± 0.008
**187**	0.3997 ± 0.0442	0.0133 ± 0.0096
**199**	0.3703 ± 0.04504	0.00615 ± 0.0059
**205**	0.648 ± 0.0687	1.263 ± 0.875
**206**	1.167 ± 0.0706	8906.87 ± 6239.617
**213**	0.068 ± 0.072	<< 1 fg/mL (Blank)
**214**	1.141 ± 0.0691	5765.294 ± 3995.229
**220**	0.669 ± 0.086	1.804 ± 1.394

* ± value is the standard deviation.

All patient urine samples except for P50, P159, and P213 showed observable LAM concentrations above 1 fg/mL. P50 and P159 exhibited SM count values marginally below the 1 fg/mL control sample while P213 was significantly below 1 fg/mL. It should be noted that P50 and P159 were not blank, instead exhibiting so few single molecule blinking events that any concentration derived from them would be less than 1 fg/mL. Meanwhile, P213 displayed negligible fluorescence and, instead, could be treated as ‘blank’. The extremely low number of molecules observed in the P213 sample is attributed to nonspecific binding. P146 was the only patient sample with SM counts that suggested a LAM concentration above 10 ng/mL. The remaining 16 patient samples had varying concentrations of LAM and did not exhibit any signs of well plate leakage, corrosion, or show evidence of nonspecific binding. Additionally, all sample wells displayed relatively uniform readings between replicates except for the third well of samples P89 and P128. These two samples had a large amount of white-colored sediment, which precipitated rapidly after vortexing, but which was present in the final 100 μL aliquot taken from each vial and is believed to be the source of increased LAM in the associated wells. Further, sample P214 was tinged with red, possibly due to blood contamination during sample collection, and also produced SM counts suggesting a high LAM concentration (5.7 ng/mL).

## Discussion

Overall, SM FLISA demonstrated good qualitative agreement with GeneXpert and culture testing results. P50 and P159 generated urine samples that produced SM counts indicative of LAM concentrations below 1 fg/mL, suggesting this concentration as a good benchmark for declaring a patient negative for TB disease. Meanwhile, 17/18 TB disease positive patients produced urine LAM concentrations appreciably above the 1 fg/mL benchmark. However, one confirmed TB patient, P213, did not produce a urine sample with appreciable LAM concentration, despite having a ‘High’ or ++++ semi-quantitative Xpert result on the associated sputum sample. With a benchmark of 1 fg/mL LAM concentration for positive declaration, this patient may have gone undetected unless clinical signs and symptoms had indicated the presence of TB disease. So far, the presence of interfering analytes has not been studied in this detection scheme and may shed light on the mismatching results with this sample. It is also important to note the apparent lack of correlation between the semi-quantitative Xpert results and SM FLISA results, namely, that high or low amounts of TB DNA observed by Xpert does not appear to correlate to respectively high or low quantities of LAM in the urine.

During the evaluation of an ultrasensitive detection method for LAM, we found that FLISA sensitivity could be extended to SM concentrations (<10 fM) using plasmonic gratings as the sensor substrate. This level of sensitivity is significant for patients that exhibit low concentrations of LAM, including patients that have either early stage or less advanced disease and may open new paths for treatment monitoring. In the first case, improved diagnosis can enable the ability to begin treatment earlier, which can reduce disease progression and transmission. In the latter case, the ability to accurately and rapidly determine if a patient is on an efficient or failing therapy, may provide clinicians with a more accurate timeline for treatment cessation, prolongation or adjustment with significantly lower risk to develop drug resistance.

Economical plasmonic gratings carry enormous potential to improve FLISA sensitivity in a host of detection schemes due to three important factors. First, exchanging capture/detection antibody pairs to target other biomarkers allows the conversion of nearly any available ELISA kit into a single molecule FLISA. Second, the inexpensive microcontact lithography manufacturing process means plasmonic gratings no longer require cost-prohibitive electron beam or interference lithography techniques. Third, the alumina capping layer eliminates or significantly reduces the corrosion problem associated with using silver in complex liquid media, allowing the higher-enhancement, lower-loss plasmonic material to replace gold in biological assays. In this work, the plasmonic gratings were integrated with detachable incubation wells through 24 well slide adapter. In the future, gratings could be integrated as the base of larger well plates (e.g. 96, 384, etc.) to improve the sensitivity of high throughput assays.

The application of the plasmonic grating platform to TB LAM demonstrates its power to dramatically improve LODs and, therefore, clinical performance of the assay. This assay, with a clinical LOD of 1fg/mL constitutes a breakthrough for the field and is the most sensitive method reported. Though this format has not yet been adapted to the point-of-care, the use of this TB LAM SM FLISA could be used as a reference assay to determine the concentration LAM in patient samples for the various clinical and research purposes outlined above.

## Supporting information

S1 Fig Method of Image Analysis. Process to determine the number of LAM molecules located within a 6 μm × 6 μm grid. The given average and standard deviation for each patient derive from counting ~12 separate grids.
(TIF)
