# Supplementary figures and images for: Ultrasensitive detection of lipoarabinomannan with plasmonic grating biosensors in clinical samples of HIV negative patients with tuberculosis
Source: PLoS One. 2019 Mar 26;14(3):e0214161. doi: 10.1371/journal.pone.0214161 (PMC6435140; doi:10.1371/journal.pone.0214161)

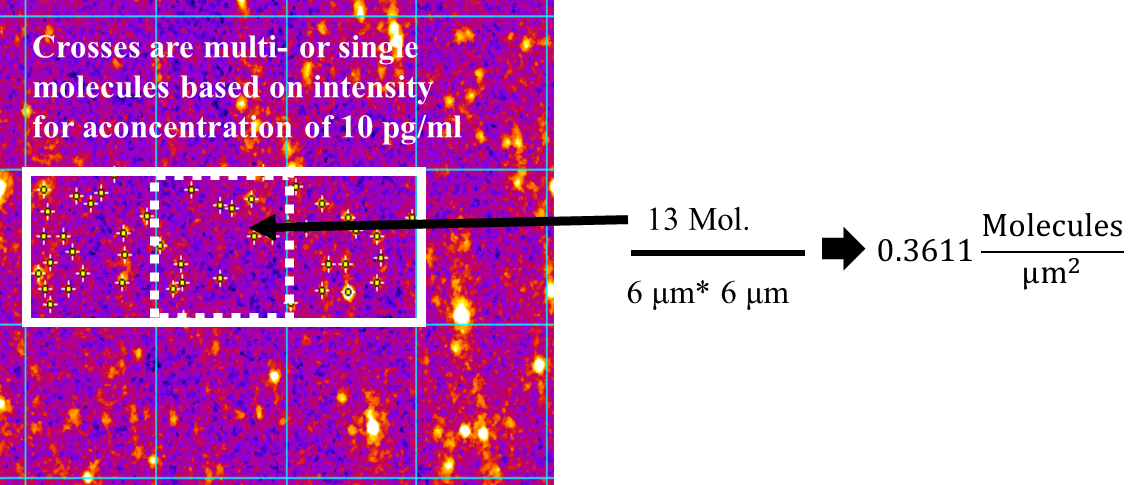

Supplement: S1 Fig — Process to determine the number of LAM molecules located within a 6 μm × 6 μm grid. The given average and standard deviation for each patient derive from counting ~12 separate grids. (TIF) [file pone.0214161.s001.tif]
